# The Mre11 Protein Is Necessary for DNA Damage Response

**DOI:** 10.1371/journal.pbio.0020144

**Published:** 2004-05-11

## Abstract

xx

With billions of cells in the adult human body, all replicating and dividing in an environment laden with toxins, radiation, and free radicals, a certain amount of DNA damage is guaranteed to occur. Fortunately, all organisms have built-in checkpoints throughout the cell cycle that prevent such mistakes from propagating. At the G1 checkpoint during cell division, for example, molecules survey nuclear DNA for errors and breaks before the cell is deemed fit to undergo S phase, the DNA replication stage. If damage is found, enzymes either work to repair it or, in some cases, trigger programmed cell death, or apoptosis. But when checkpoints fail, and DNA damage is left unrepaired, disease such as cancer can result. A better understanding of these events, as provided, for example, by Vincenzo Costanzo and colleagues in this issue, will consequently lead to a better understanding of the mechanisms that give rise to cancer.[Fig pbio-0020144-g001]

**Figure pbio-0020144-g001:**
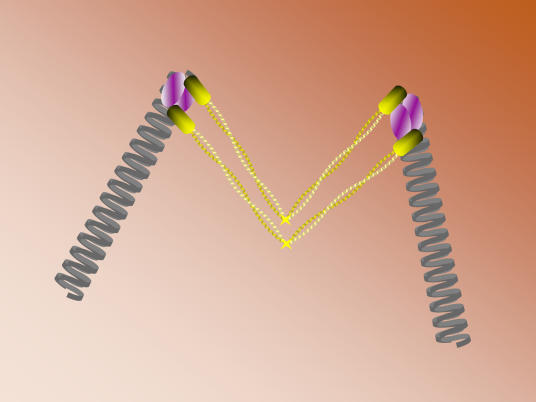
Model for Mre11 complex bridging DNA molecules

A serious form of DNA damage, called a double strand break (DSB), cuts the helix clean through—a far worse scenario than if just one strand slips free. In response to a DSB, the cell recruits a signaling protein called ATM and a three-protein complex called MRN, whose components selectively bind to broken DNA ends. A malfunction of this signal and repair pathway is dire. People who suffer from the genetic disease ataxia-telangiectasia (A-T) lack a functioning ATM molecule and therefore cannot properly handle DSBs or successfully navigate the G1 checkpoint. This condition leads to a host of problems, including abnormal chromosomes, deficient immune function, and a predisposition to cancer. A-T-like disease (ATLD), another rare genetic condition, has very similar symptoms. The only difference is that the protein missing is Mre11, a subunit of MRN. While recent work on the cellular level has indicated that MRN activates ATM, the biochemical relationship between these proteins has yet to be fully understood.

Studying these two molecules using traditional biochemical assays is difficult because knocking out the activity of these proteins is lethal to many cells. Costanzo and colleagues used a novel test system of cell-free frog extracts and found that Mre11 is necessary for both ATM activation and for the formation of large protein–DNA complexes apparently responsible for triggering the cascade of signaling molecules underlying the DNA damage response at the G1 checkpoint.

The frog extract system allowed the team to manipulate the presence or absence of Mre11 and accurately measure the response triggered by the addition of fragmented bits of DNA (simulating naturally occurring DSBs). As predicted, without a functional Mre11 protein, ATM was not activated and there was no response. By simply adding the protein Mre11 back to the mixture, the damage response was restored. But when the researchers added a mutant form of Mre11, still capable of performing its essential tasks in another stage of the cell cycle—DNA replication—the G1 damage response remained suppressed. This mutant form of the Mre11 protein lacks the C-terminal, or DNA-binding, end.

Costanzo and colleagues also found that this DNA-binding end is required for the assembly of DNA–ATM–MRN complexes in the presence of fragmented DNA and seems to direct the entire damage response. This work helps to explain the similarity between patients with A-T and those with ATLD, and hints at the formation of a large “signaling” complex that helps to orchestrate the crucial response to DBSs in DNA.

